# Motor learning principles in the service of speech disorders

**DOI:** 10.1192/j.eurpsy.2022.2243

**Published:** 2022-09-01

**Authors:** E. Vashdi

**Affiliations:** Yaelcenter institute, Clinic, Aloney Aba, Israel

**Keywords:** Motor learning principles, Apraxia of speech, autism, VML method

## Abstract

**Introduction:**

Childhood Apraxia of Speech (CAS) was declared as a motor speech disorder by ASHA (2007). Yet, until then it was mainly addressed as a phonological disorder and until these days, 14 years later, the treatment of CAS is yet to be motor based worldwide. Professionals finds it hard to diagnose it clearly due to comorbidity with communication and language disorders.

**Objectives:**

This non clarity might lead to non-accurate treatment since the essence of the syndrome is not addressed. An accurate treatment will integrate knowledge from several domains: communication, Language, Sensory, behavioural, emotional, cognitive and, the most important one for CAS, motor learning.

**Methods:**

Motor learning is an area of knowledge which is learnt usually in sport academy, while Its main practical purpose is to improve training methods in sport. The use of motor learning knowledge doesn’t belong to the world of sport primarily but rather to the world of movement wherever it exists. One of the fascinating areas of movement is speech.

**Results:**

Speech in its basic form is motor based, before it being used as a motor tool for language and communication. It is the most complicated motor task in the human body since for every syllable we activate directly and indirectly over 100 muscles. The children who can’t acquire speech spontaneously due to severe deficit in motor planning, need to practice motor speech tasks repeatedly and accurately.

**Conclusions:**

This lecture will present the use of 20 motor learning principles in the speech treatment via the VML method

**Disclosure:**

I am the founder of the VML method while teaching it in various countries

